# Emerging trends of the tumor microenvironment in peritoneal malignancies (2010-2024): a visualization analysis

**DOI:** 10.3389/fonc.2025.1515476

**Published:** 2025-06-04

**Authors:** Hua Duan, Mengqi Cheng, Qianhui Sun, Cihui Chen

**Affiliations:** ^1^ Department of Oncology, The First Affiliated Hospital of Zhejiang Chinese Medical University (Zhejiang Provincial Hospital of Chinese Medicine), Hangzhou, China; ^2^ Cancer Institute of Integrated Traditional Chinese & Western Medicine Zhejiang Chinese Medical University, Hangzhou, China

**Keywords:** tumor microenvironment, peritoneal malignancies, immunotherapy, tumor-associated macrophages, hyperthermic intraperitoneal chemotherapy, cytoreductive surgery, cancer-associated fibroblast, visualization analysis

## Abstract

**Background:**

Peritoneal malignancies (PM) represent a group of highly heterogeneous tumors associated with poor prognosis and limited effective treatment options. Recent studies have demonstrated significant progress in understanding the tumor microenvironment (TME) of PM. However, no bibliometric analysis focusing on PM and TME has been conducted. This study aims to provide a comprehensive assessment of the current research landscape and to identify key areas of interest and emerging trends in this field from a bibliometric perspective.

**Methods:**

Publications related to the TME in PM from 2010 to 2024 were retrieved from the Web of Science Core Collection database. Microsoft Excel, VOSviewer, CiteSpace, and R package “bibliometrix” were used to perform the visualization analysis.

**Results:**

A total of 862 papers from 56 countries were included. Both annual publication counts and citations have increased steadily over time. The United States of America (USA) contributed the highest number of publications and demonstrated the greatest impact, followed by China and Japan. The University of Texas MD Anderson Cancer Center, Sichuan University and Fudan University were identified as the leading research institutions. Four of the top five most prolific authors are from Japan, including Kajiyama Hiroaki, Yashiro Masakazu, Fushida Sachio and Kinoshita Jun. *Cancers* published the largest number of articles, with 56 publications, while *Cancer research* was the most frequently co-cited journal. Reference and keyword burst detection revealed that research hotspots include cytoreductive surgery, hyperthermic intraperitoneal chemotherapy, immunotherapy, tumor-associated macrophages, cancer-associated fibroblast and endothelial growth factor.

**Conclusions:**

This study summarizes recent research frontiers and hotspots regarding the TME in PM and provided valuable references for future investigations. Immunotherapy targeting the TME is likely to become a major research direction.

## Introduction

Peritoneal malignancies (PM) represent a group of highly heterogeneous tumors, including primary peritoneal tumors and secondary peritoneal metastases, such as digestive tract tumors, gynecological cancers, sarcomas, as well as rare extraperitoneal tumors, such as lung, kidney, and breast cancers (1).The Global Cancer Observatory (GLOBOCAN) provides global cancer incidence estimates for 185 countries but does not report the incidence of PM separately (2), likely due to the difficulty of detecting PM through cross-sectional imaging. It is generally believed that the incidence of PM correlates with that of primary tumors, with stomach and ovarian cancer showing the highest rates (3). Statistics indicate that about 70% of patients with PM present with ascites, often accompanied by omental and mesenteric infiltration and peritoneal nodules. PM has long been regarded as an advanced malignancy with limited treatment options and an extremely poor prognosis. Over the past few decades, treatment advances, particularly cytoreductive surgery (CRS) combined with intraperitoneal chemotherapy, have significantly prolonged survival in carefully selected patients (4). The TME contains a variety of cell and cytokines that can promote or inhibit tumor progression. The peritoneum, divided into visceral and parietal layers, forms a continuous membrane covering the internal organs, mesentery, abdominal wall and pelvic cavity (5). It facilitates the exchange of nutrients, metabolites, and gases and is rich in innate and adaptive immune cells, cytokines and chemokines. Increasing evidence suggests that, beyond the standard CRS and intraperitoneal chemotherapy, therapies targeting the TME has achieved remarkable efficacy, including promoting anti-tumor immunity and inhibiting angiogenesis, warranting further attention.

This study employed bibliometric methods to analyze articles related to TME in PM from 2010 to 2024, identifying research hotspots through statistical and data visualization techniques.

## Methods

### Data source and search strategy

**Figure 1:**
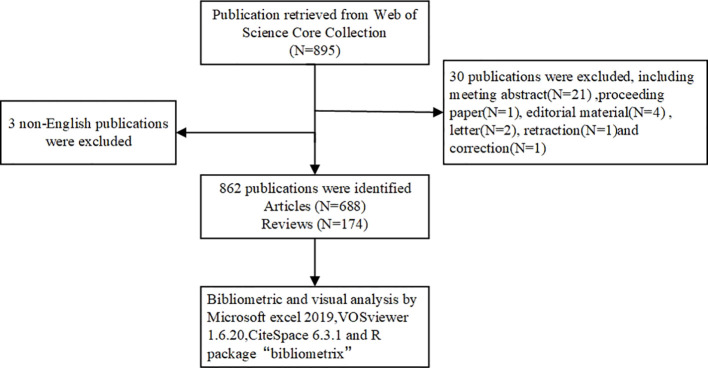
Flowchart of the screening process on research of TME in PM.

The Web of Science Core Collection (WOSCC) was selected to search for relevant literature. Search terms were developed using synonyms from Medical Subject Headings (MeSH) and related textual terms. Follow the search strategy below: TS= (“peritoneal malignancies” OR “peritoneal cancer” OR “peritoneal malignancies” OR “peritoneal neoplasms” OR “peritoneal malignancy” OR “peritoneal tumor”) AND (“tumor microenvironment” OR “cancer microenvironment”). Further screening was conducted based on the following criteria: (1) publication date between January 1, 2010 and August 12, 2024; (2) language restricted to English to avoid bias from multilingual translations; and (3) document type limited to articles and reviews. The processes of data collection, selection, and extraction were independently conducted by two authors and subsequently cross-validated. Discrepancies during the research process were resolved through discussion or consultation with CH Chen. All relevant literature was retrieved and downloaded in “Plain text” format to minimize potential biases caused by database updates. The content of the records and the cited references were comprehensively documented. All valid data were imported to CiteSpace and deduplicated for subsequent visual analysis. The retrieval process is illustrated in **Figure 1**.

### Data collection and bibliometric analysis

Microsoft Office Excel 2019 was used to calculate the total number of annual publications, average citations, annual publications by country, cumulative publications by institutions, journals and authors, as well as the impact factor (IF) and H-index. The research overview, hot spots and trends were obtained using bibliometric methodology (6). VOSviewer, a widely recognized bibliometric analysis software, can generate maps of publications categorized by countries, institutions, journals, and authors. The size, color, and thickness of node connections on the map indicate various relationships and levels of collaboration. CiteSpace (version 6.3.1), another bibliometric analysis software, was used to draw a dual-map overlay and to analyze references using Citation Bursts (7). The R package “bibliometrix” (version 4.3.0) was used to create a global distribution network of publications of TME of PM.

## Results

In this study, ethical approval was not required, as the data were obtained from publicly accessible databases and did not involve new studies with human or animal subjects.

### Trends of publication outputs, citations and H-index

**Figure 2:**
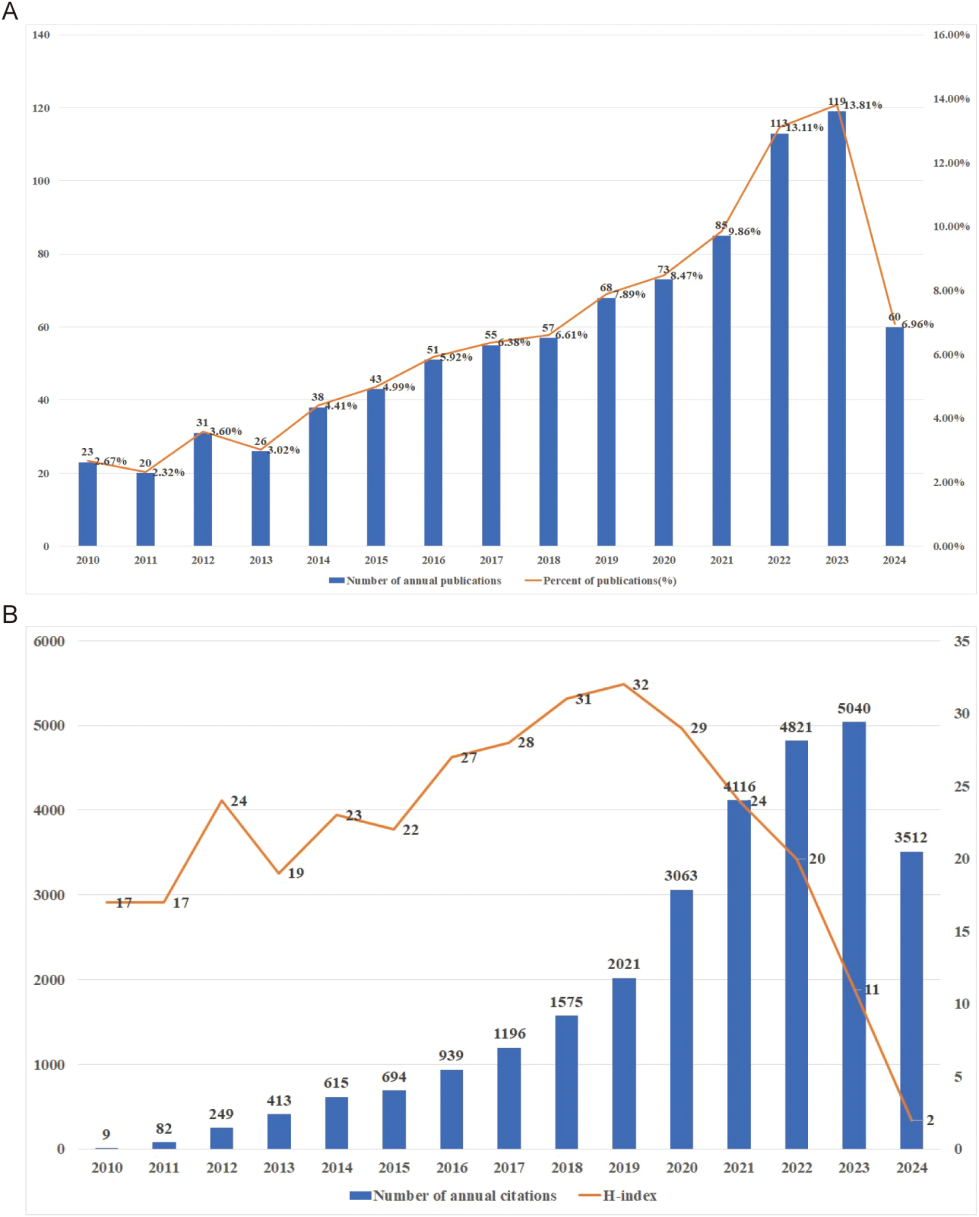
**(A)** Publications on research of TME in PM. **(B)** Citations and H-index on research of TME in PM.

Based on the established search strategy, a total of 862 studies were included, comprising 688 articles and 174 reviews related to the TME in PM. **Figure 2** showed the basic characteristics of the 862 publications. **Figure 2A** illustrates that the number of publications increased steadily from 2010 to 2024, with minor decreases in 2011 and 2013. To date, the 862 publications have received a total of 23,352 citations, resulting in an average citations per publication (CPP) of 32.89. Consistent with the publication trend, the number of annual citations has also gradually increased, peaking at 5,040 citations in 2023. In 2021 and 2022, the number of citations surpassed 4,000, reaching 4,116 and 4,821 citations respectively, as shown in **Figure 2B**. The H-index of all 862 publications was 82. Specifically, the H index showed a trend of first rising and then falling, with the highest in 2019 which was 32. It is important to note that the figures for publications, annual citations and H-index in 2024 do not represent the complete totals for the entire year.

### Analysis of countries

**Figure 3:**
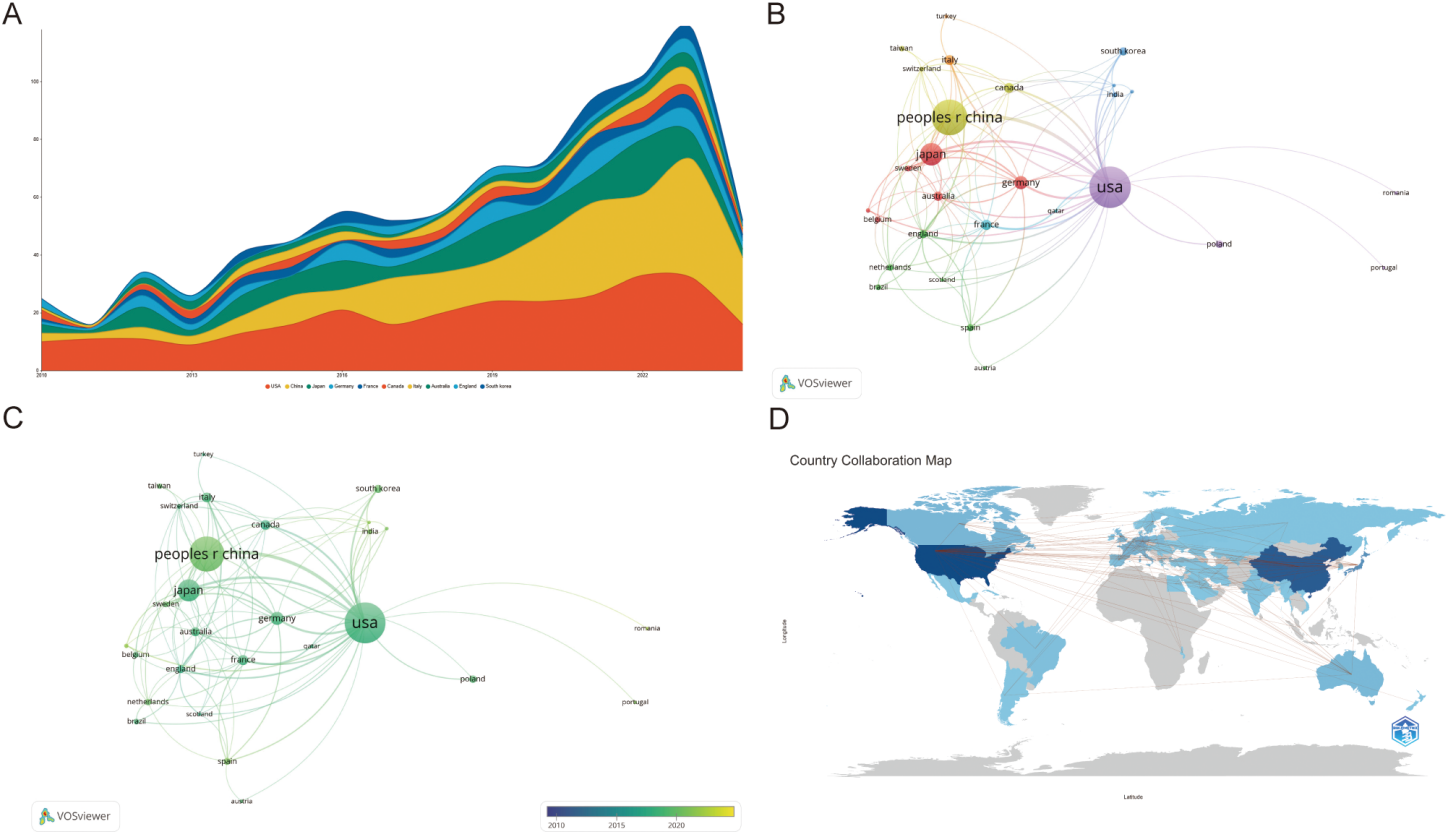
**(A)** Top 10 countries on research of TME in PM. **(B, C)** The visualization of countries. The size of the nodes indicates the number of publications, while the thickness and length of the connections illustrate the strength and significance of their relationships. **(D)** The geographical distribution generated by bibliometrix.

Publications on the TME in PM originated from 56 countries. **Table 1** presents the characteristics of the top ten countries, including four from Europe, three from Asia, two from North America, and one from Oceania. The USA, China and Japan contributed the highest proportion of publications, accounting for 32.60%, 26.22%, and 13.11%, respectively. Together, China, the USA and Japan produced nearly three-quarters of the total publications (73.93%). The H-index, total link strength (TLS), and total citations were largely consistent with the number of publications. Research from the USA, China and Japan was relatively more in-depth and recognized (**Figure 3A**). Strong links were observed among different countries, indicating some overlap in published content. Countries with five or more publications were visualized, and a collaborative network was constructed, revealing close cooperation and exchanges. The USA exhibited the most frequent collaborations with China, Japan, Australia, Canada and so on. China also maintained active collaborations, followed by Japan (**Figures 3B–D**).

**Table 1:** Top 10 countries on research of TME in PM.

Rank	Country	Article count	Percentage (n/862)	Citations	Average citation per article	Total link strength	H-index
1	USA	281	32.60%	11019	39.21	132	54
2	China	226	26.22%	6036	26.71	48	40
3	Japan	113	13.11%	4739	41.94	45	35
4	Germany	53	6.15%	2634	49.70	47	27
5	France	36	4.18%	1041	28.92	26	18
6	Canada	33	3.83%	1022	30.97	19	20
7	Italy	33	3.83%	1256	38.06	21	18
8	Australia	30	3.48%	1231	41.03	23	19
9	England	26	3.02%	1463	56.27	32	16
10	South Korea	25	2.90%	490	19.60	12	10

### Analysis of institutions

**Figure 4:**
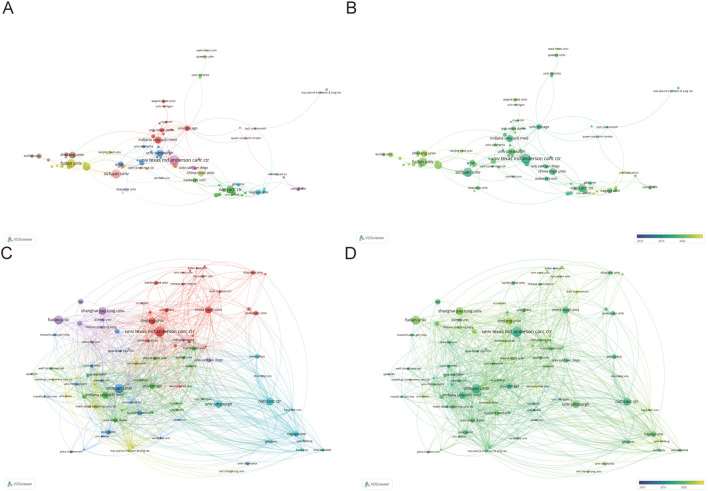
**(A, B)** Institutions’ collaboration visualization. **(C, D)** Institutions’ citation visualization. The size of the nodes indicates the number of publications, while the thickness and length of the connections illustrate the strength and significance of their relationships.

1291 institutions have published articles of the TME in PM. Given that the number of publications is consistent across multiple institutions, we have counted the top 13 institutions, which are shown in **Table 2**. And the top 13 institutions are located in China, the USA and Japan, with six of them based in China. The University of Texas MD Anderson Cancer Center has the highest publication count with 22 papers, followed by Sichuan University with 20 papers and Fudan University with 19 papers. National Cancer Center occupied the highest contribution of citations and average citation (57.41) while The University of Texas MD Anderson Cancer Center had the highest H-index which was 15. **Figure 4** illustrates the strong cooperation among various institutions, including The University of Texas MD Anderson Cancer Center, Indiana University School of Medicine, and University of Pittsburgh.

**Table 2:** Top 13 institutions on research of TME in PM.

Rank	Institutions	Country	Article count	Percentage (n/862)	Citations	Average citation per article	Total link strength	H-index
1	The University of Texas MD Anderson Cancer Center	USA	22	2.55%	816	37.09	19	15
2	Sichuan University	China	20	2.32%	823	41.15	4	12
3	Fudan University	China	19	2.20%	391	20.58	15	10
4	National Cancer Center	Japan	17	1.97%	976	57.41	15	12
5	Shanghai Jiao Tong University	China	17	1.97%	930	54.71	18	10
6	China Medical University	China	15	1.74%	417	27.80	6	10
7	Indiana University School of Medicine	USA	15	1.74%	697	46.47	18	13
8	University of Pittsburgh	USA	15	1.74%	390	26.00	12	6
9	Zhejiang University	China	15	1.74%	282	18.80	7	7
10	University of Chicago	USA	12	1.39%	684	57.00	17	9
11	Huazhong University of Science Technology	China	12	1.39%	144	12.00	4	7
12	Nagoya University	Japan	12	1.39%	570	47.50	18	9
13	NIH National Cancer Institute	USA	12	1.39%	260	21.67	8	9

### Authors and co-cited authors

**Figure 5:**
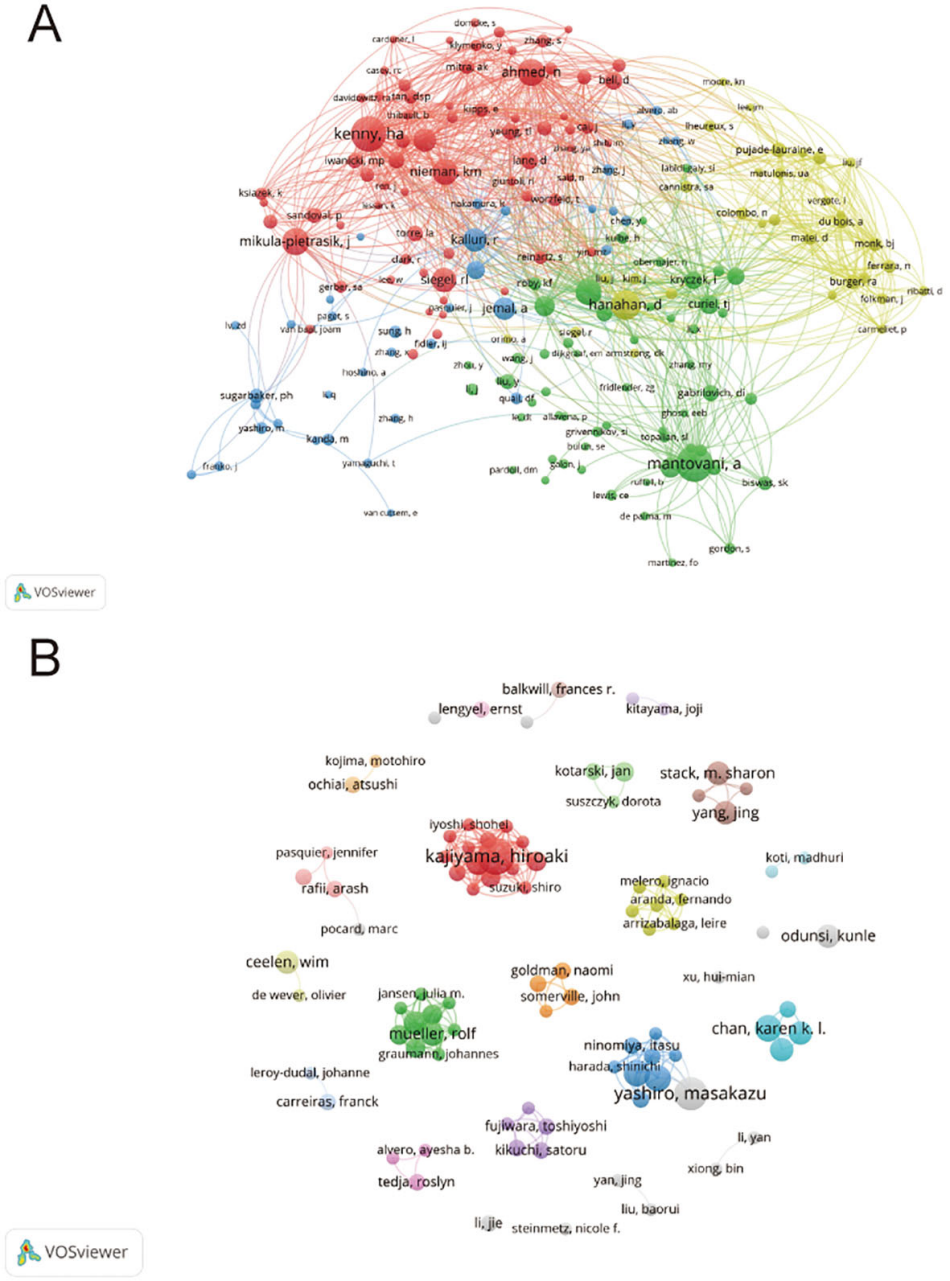
**(A)** Authors’ collaboration visualization. **(B)** Authors’ co-citation visualization. The size of the nodes indicates the number of publications, while the thickness and length of the connections illustrate the strength and significance of their relationships.

Authors and co-cited authors were analyzed to identify leading scholars and influential contributors in the field. A total of 5,914 authors contributed to 862 articles on the TME in PM. Due to ties in publication counts, the top 14 authors are listed in **Table 3**. Four of the top five authors were from Japan, including Kajiyama Hiroaki (11 articles), Yashiro Masakazu (10 articles), Fushida Sachio (8 articles), and Kinoshita Jun (8 articles). They also achieved the highest H-index (nine, eight or seven). The highest average number of citations(62.86) was recorded by Müller Rolf and Reinartz Silke from Germany. A collaborative network was also established for authors with four or more published articles. Visual analysis revealed close collaborations, particularly among Kajiyama Hiroaki, Yashiro Masakazu, Karen K. L. Chan, and Fushida Sachio (**Figure 5A**). Among 31,744 co-cited authors, 22 were cited more than 50 times. Specifically, Mantovani, A is the most co-cited author (150 times), followed by Kenny H.A. (143 times) and Hanahan D (114 times). Two authors had TLS exceeding 2000, namely Kenny H.A. (TLS=2852) and Nieman K.M. (TLS =2249). Positive collaborations were also evident among different co-cited authors (**Figure 5B**).

**Table 3:** Top 14 authors on research of TME in PM.

Rank	Author	Country	Article count	Percentage (n/862)	Citations	Average citation per article	Total link strength	H-index
1	Kajiyama, Hiroaki	Japan	11	1.28%	455	41.36	65	9
2	Yashiro, Masakazu	Japan	10	1.16%	219	21.90	6	8
3	Karen K L Chan	China	8	0.93%	307	38.38	19	6
4	Fushida, Sachio	Japan	8	0.93%	177	22.13	36	7
5	Kinoshita, Jun	Japan	8	0.93%	177	22.13	36	7
6	Ceelen, Wim	Belgium	7	0.81%	172	24.57	3	6
7	Müller, Rolf	Germany	7	0.81%	440	62.86	19	6
8	Ngan, Hextan Y. S.	China	7	0.81%	305	43.57	0	7
9	Odunsi, Kunle	USA	7	0.81%	386	55.14	72	5
10	Reinartz, Silke	Germany	7	0.81%	440	62.86	45	6
11	Stack, M. Sharon	USA	7	0.81%	177	25.29	11	7
12	Yang, Jing	USA	7	0.81%	200	28.57	10	3
13	Yoshihara, Masato	Japan	7	0.81%	151	21.57	53	5
14	Yung, Mingo M. H.	China	7	0.81%	305	43.57	19	7

### Journals and co-cited journals

**Figure 6:**
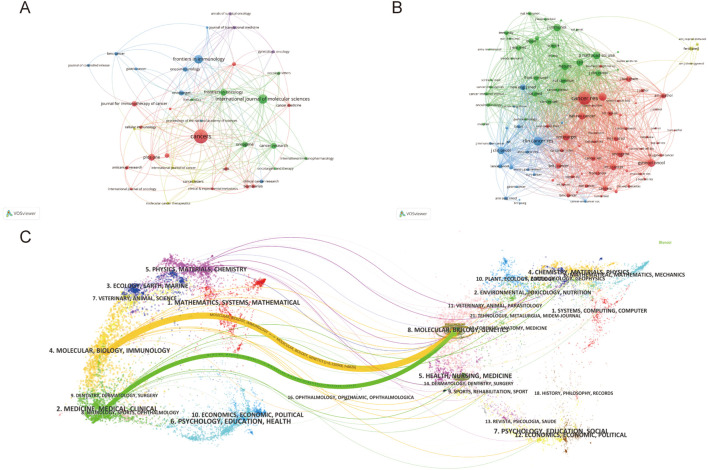
**(A)** Journals’ collaboration visualization. **(B)** Journals’ co-citation visualization. The size of the nodes indicates the number of publications, while the thickness and length of the connections illustrate the strength and significance of their relationships. **(C)** Dual-map overlay of journals. The left label denotes citing journals, the right label denotes cited journals, and the colored paths illustrate the citation relationships between them.

The study retrieved articles published in 345 journals, with details of the top 10 journals presented in **Table 4**. The top three journals are *Cancers* (6.5% of 862 articles, 56 papers), *International Journal of Molecular Sciences* (3.6% of 862 articles, 31 papers), and *Frontiers in Immunology* (3.25% of 862 articles, 28 papers). *Oncogene* has the highest average citation (98.47), followed by *Cancer Research* (83.25 average citations). *Cancer Research* (IF=12.5) was the journal with the highest IF, and *Journal for Immunotherapy of Cancer* (IF=10.3) ranked second. *Cancer* has the highest H-index at 21, followed by *Plos On*e with an H-index of 17. We visually analyzed 39 journals with at least five articles and plotted the network (**Figure 6**). **Figure 6A** displays six clusters and 181 links among the journals. *Cancer* has close citation relationships with journals such as *International Journals of Molecular Sciences*, *International Immunopharmacology*, and *Plos One*. There are also close links between other magazines, including *Frontiers in immunology*, *Frontiers in oncology*, etc. **Table 5** lists the top ten collectively cited journals, with eight are from the USA and two are from the England. *Cancer research* (n=2355) and *Clinical Cancer Research* (n=1275) are two journals with more than 1,000 citations. The co-citation network map in **Figure 6B** illustrates journals with a minimum of 100 co-citations.

**Table 4:** Top 10 journals on research of TME in PM.

Rank	Journal Title	Country	Count	Percentage (n/862)	Citations	Average citation per article	Total link strength	H-index	IF (2023)	Quartile in category (2023)
1	*Cancers*	Switzerland	56	6.50%	1432	25.57	141	21	4.5	Q1
2	*International Journal of Molecular Sciences*	Switzerland	31	3.60%	618	19.94	82	13	4.9	Q1
3	*Frontiers in Immunology*	Switzerland	28	3.25%	349	12.46	29	11	5.7	Q1
4	*Plos One*	USA	20	2.32%	722	36.10	26	17	2.9	Q1
5	*Frontiers in Oncology*	Switzerland	19	2.20%	369	19.42	63	8	3.5	Q2
6	*Cancer Research*	USA	16	1.86%	1332	83.25	38	15	12.5	Q1
7	*Oncogene*	England	15	1.74%	1477	98.47	54	14	6.9	Q1
8	*Journal for Immunotherapy of Cancer*	England	14	1.62%	227	16.21	12	7	10.3	Q1
9	*Oncotarget*	USA	13	1.51%	693	53.31	23	12	5.168	/
10	*Oncoimmunology*	USA	12	1.39%	839	69.92	27	11	6.5	Q1

**Table 5:** Top co-cited 10 journals on research of TME in PM.

Rank	Journal Title	Country	Citations	Total link strength	IF (2023)	Quartile in category (2023)
1	*Cancer Research*	USA	2355	251771	12.5	Q1
2	*Clinical Cancer Research*	USA	1275	144188	10.1	Q1
3	*Plos One*	USA	997	106121	2.9	Q1
4	*Gynecologic Oncology*	USA	993	123880	4.5	Q1
5	*Proceedings of the National Academy of Sciences of the United States of America*	USA	970	97958	9.4	Q1
6	*Nature*	England	958	104491	50.5	Q1
7	*Journal of Immunology*	USA	931	77414	3.6	Q2
8	*Journal of Clinical Onocology*	USA	878	103462	42.1	Q1
9	*Cell*	USA	835	85815	45.5	Q1
10	*Nature Reviews Cancer*	England	821	84599	72.5	Q1

**Figure 6C** presents a dual-map overlay encompassing all journals. There are one green path and one orange path which represented different citation paths. The orange path indicates that Molecular, Biology, and Immunology are the main fields of articles included. The green path indicates Medicine, Medical and Clinical are the main fields. Interestingly, the fields of Molecular, Biology and Genetics cover most of the cited articles, both in the orange and green paths.

### Analysis of co-cited references and reference with citation bursts

**Figure 7:**
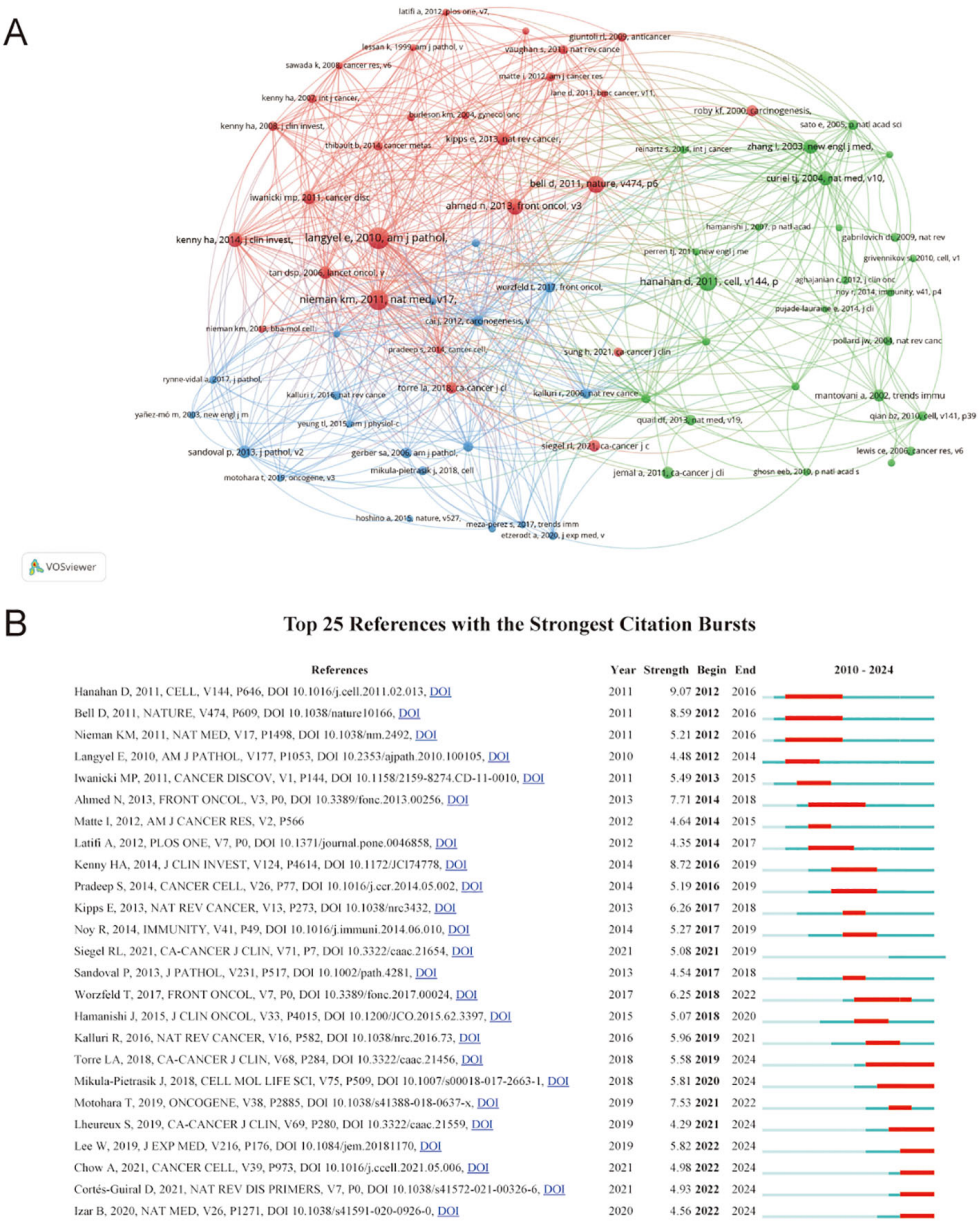
**(A)** Visualization of co-cited references. The size of the nodes indicates the number of publications, while the thickness and length of the connections illustrate the strength and significance of their relationships. **(B)** Top 25 references with the strongest citation. The red bar indicates the time interval when the reference co-citation burst started and ended.

The analysis of co-cited references identified the most influential publications and helped generalize research hotspots. A total of 43854 co-cited references were recorded for TME in PM. Due to ties in citation counts across multiple journals, **Table 6** presents the key features of the 12 most highly cited references. The most frequently cited reference was “*Ovarian cancer development and metastasis*” published in *American Journal of Pathology*, with 80 citations, followed by Kristin M Nieman’s article “*Adipocytes promote ovarian cancer metastasis and provide energy for rapid tumor growth*” published in Nature Medicine with 73 citations. **Figure 7A** illustrates close links among references cited at least 20 times.

**Table 6:** Top 12 related articles with the most citations on research of TME in PM.

Rank	References	Journal	IF (2023)	First author	Publication time	Citations
1	Ovarian cancer development and metastasis	*American Journal of Pathology*	4.7	Ernst Lengyel	2010	80
2	Adipocytes promote ovarian cancer metastasis and provide energy for rapid tumor growth	*Nature Medicine*	58.7	Kristin M Nieman	2011	73
3	Hallmarks of cancer: the next generation	*Cell*	45.5	Douglas Hanahan	2011	64
4	Integrated genomic analyses of ovarian carcinoma	*Nature*	50.5	Cancer Genome Atlas Research Network	2011	58
5	Getting to know ovarian cancer ascites: opportunities for targeted therapy-based translational research	*Frontiers in Oncology*	3.5	Nuzhat Ahmed	2013	55
6	Mesothelial cells promote early ovarian cancer metastasis through fibronectin secretion	*Journal of Clinical Investigation*	13.3	Hilary A Kenny	2014	48
7	Specific recruitment of regulatory T cells in ovarian carcinoma fosters immune privilege and predicts reduced survival	*Nature Medicine*	58.7	Tyler J Curiel	2004	47
8	Intratumoral T cells, recurrence, and survival in epithelial ovarian cancer	*New England Journal of Medicine*	96.2	Lin Zhang	2003	46
9	Ovarian cancer spheroids use myosin-generated force to clear the mesothelium	*Cancer Discovery*	29.7	Marcin P Iwanicki	2011	42
10	Meeting the challenge of ascites in ovarian cancer: new avenues for therapy and research	*Nature Reviews Cancer*	72.5	Emma Kipps	2013	42
11	Carcinoma-associated fibroblasts derive from mesothelial cells via mesothelial-to-mesenchymal transition in peritoneal metastasis	Journal of Pathology	5.6	Pilar Sandoval	2013	41
12	Mechanisms of transcoelomic metastasis in ovarian cancer	Lancet Oncology	41.6	David S P Tan	2006	41

Reference with citation bursts indicate publications that were widely cited over a specific period. In this study, the earliest citation burst occurred in 2012, referencing a 2010 publication. Currently, seven references continue to exhibit active citation bursts. The reference “*Extracellular vesicles: exosomes, microvesicles, and friends*” by Hanahan D demonstrated the strongest citation burst (strength=9.07) during the period from 2012 to 2016. Among the 25 references analyzed, the lowest citation burst strength was 4.9 (**Figure 7B**).

### Keywords analysis

**Figure 8:**
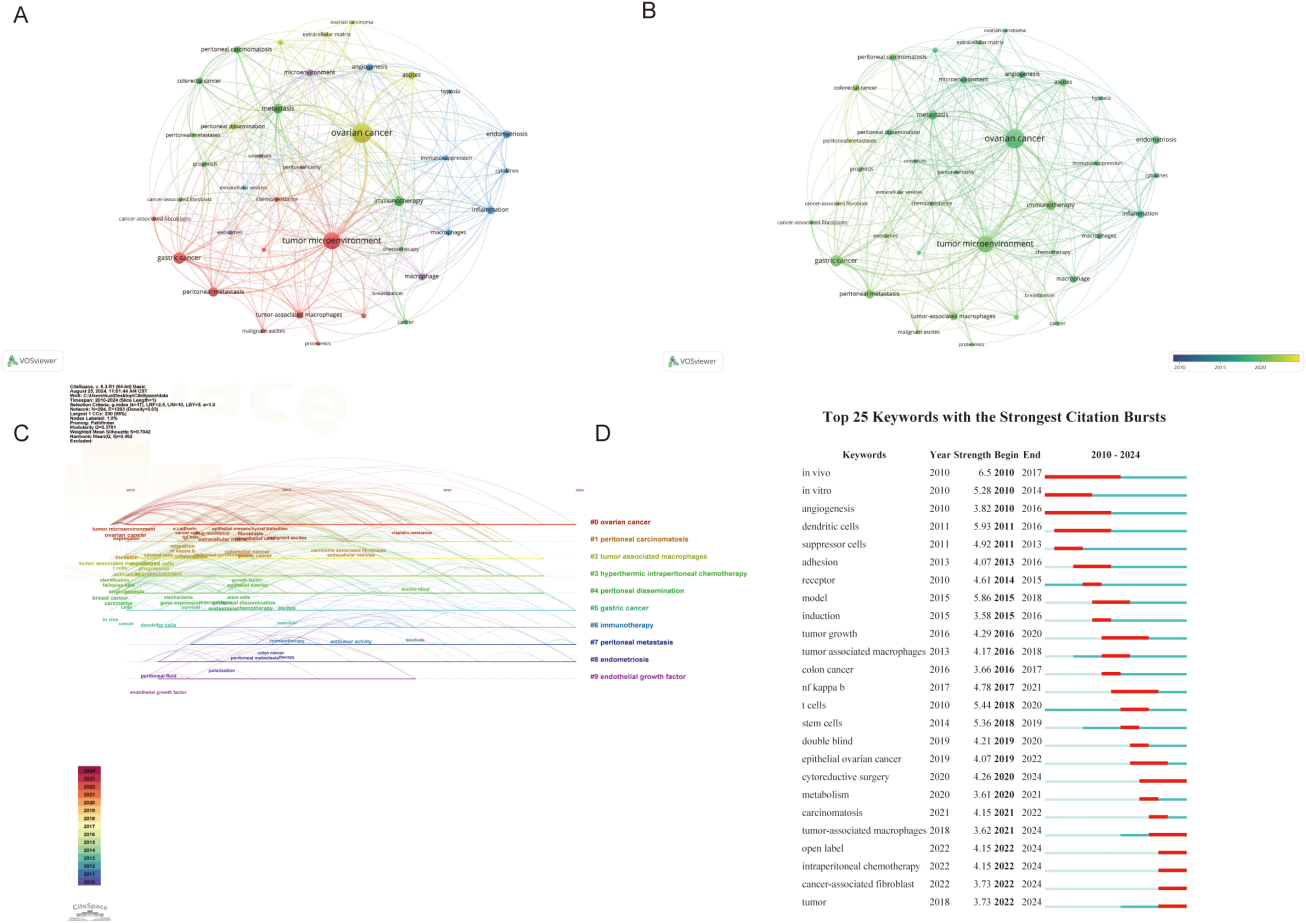
**(A, B)** Visualization of Keywords. Nodes are proportional in size to the frequency of keyword occurrence and the color of the nodes is determined by their category in cluster analysis. **(C)** Timeline view of keywords. **(D)** Top 25 keywords with the strongest citation. The red bar indicates the time interval when the reference co-citation burst started and ended.

Keywords summarize the principal concepts presented in the article. We create visual maps and perform cluster analysis for keywords appearing more than ten times. There are 300 links and six clusters for 39 keywords, represented by 6 colors. Cluster 1 contained 10 items including cancer-associated fibroblast, chemoresistance, epithelial ovarian cancer, gastric cancer, malignant ascites, pancreatic cancer, proteomics, tumor microenvironment and tumor-associated macrophages. Cluster 2 contained 9 items including cancer, cancer-associated fibroblast, chemotherapy, colorectal cancer, immunotherapy, metastasis, peritoneal carcinomatous, peritoneal metastases and prognosis. Cluster 3 contained 7 items including angiogenesis, cytokines, endometriosis, hypoxia, immunosuppression, inflammation and macrophages. Cluster 4 contained 6 items including ascites, extracellular matrix, mesothelial cells, ovarian cancer, ovarian carcinoma and peritoneal dissemination. Cluster 5 contained 6 items including breast cancer, exosomes, macrophage, microenvironment, omentum and peritoneal cavity. Cluster 6 contained 1 item including extracellular vesicles. **Figures 8A, B** illustrates the latest perspective of the TME in PM. The yellower points indicate key components, including cancer-associated fibroblasts (CAFs), prognosis, malignant ascites, epithelial, chemoresistance, tumor-associated macrophages, chemotherapy, immunotherapy and other factors.

The keyword timeline map integrates temporal and thematic dimensions, providing a dynamic representation of evolving research trends over time (**Figure 8C**). Most articles were published after 2010. It was found that “ovarian cancer” (cluster #1), and “gastric caner” (cluster #5) remained consistent hotspots from 2010 to 2024. “Endothelial growth factor” (cluster #9) had the shortest hotspot research span. By 2023, “tumor-associated macrophages”(Cluster #2), “hyperthermic intraperitoneal chemotherapy” (Cluster #3), and “peritoneal dissemination” (Cluster#4) continued to be major research hotspots. Notably, “immunotherapy”(Cluster #3), “endometriosis” (Cluster #6) and “peritoneal metastasis” (Cluster #7) emerged as the latest research hotspot in 2024.

Burst keywords are also important indicators reflecting emerging frontiers in the field. The first cited burst keywords appeared in 2010, involving keywords such as *in vivo*, *in vitro* and angiogenesis. Over time, immune cells in TME have increasingly become a research focus. Keywords such as cytoreductive surgery, tumor-associated macrophages (TAMs), open label, intraperitoneal chemotherapy and CAFs continued to emerge through 2024, indicating these topics will likely remain future research hotspots deserving high attention (**Figure 8D**).

## Discussion

### General information

From 2010 to 2024, publications of the TME in PM steadily increased, with only minor declines in 2011 and 2013, indicating strong interest among researchers. Annual citations reflect research popularity, and our study found that they have increased over time. Since 2020, annual citations have surpassed 3,000. An analysis by country shows that the USA has the highest number of publications, H-index, TLS, and total citations, indicating its leadership in this area of research. China and Japan ranked second and third, respectively. Together, they account for nearly three-quarters (73.93%) of the papers published in China, the USA and Japan, indicating that research in these three countries is more in-depth and recognized in relative terms. Cooperation and exchanges between countries are very close. The USA has a connection strength of 124, demonstrating its close cooperation with China, Japan, Germany, Australia, Canada and other countries. China similarly engages in close collaborations with numerous countries, and Japan is also actively involved in such partnerships. The top 13 institutions are located in China, the USA and Japan. The University of Texas MD Anderson Cancer Center collaborates closely with the Indiana University School of Medicine, the University of Pittsburgh, and the University of Chicago. However, it has limited institutions with institutions in China. Similarly, Fudan University engages more with domestic institutions but lacks cooperation with international ones. The above results show that establishing outstanding research institutions is crucial for enhancing research quality and that strengthening communication among different institutions is equally important.

Among the top five authors, four are from Japan and one is from China: Kajiyama Hiroaki (11 articles), Yashiro Masakazu (10 articles), Fushida Sachio (8 articles), Kinoshita Jun (8 articles) and Karen K. L. Chan (8 articles). Kajiyama Hiroaki, Yashiro Masakazu, Karen K. L. Chan, Fushida Sachio had more connections with other authors. As shown in **Table 4**, the current journals with the most research publications are *Cancer*(56 articles), *International Journal of Molecular Sciences* (31 articles), and *Frontiers in Immunology* (28 articles). The journals with the highest IF were *Cancer Research* (IF=12.5), followed by *Journal for Immunotherapy of Cancer* (IF=10.3), with all journals having an IF between 2.9 and 12.5. Most of these journals are classified in the JCR Q1 region, indicating their high research value in this field. Among the co-cited journals, most high-impact Q1 journals offer robust evidence for TME research related to PM. All journals having an IF between 2.9 and 72.5. This range of impact factors demonstrates the overall quality of research published in these journals.

### Knowledge base

Co-cited literature serves as the foundational research in a specific field. The 13 most cited articles form the foundation of the TME field in PM. Except for “*Hallmarks of cancer: the next generation*, “, a 2011 review in *Cell* by Professor Douglas Hanahan, the remaining 12 papers focus on ovarian cancer. Research interests encompass the mechanisms by which adipocytes, mesothelial cells, tumor-associated fibroblasts and regulatory T cells promoting peritoneal metastasis of ovarian cancer, as well as the treatment of ovarian ascites and genomic analysis of the disease.

References with a citation burst are those frequently cited by researchers recently, indicating an emerging topic in the field. We observed that the literature with citation bursts first appeared in 2012, published by Ernst Lengyelin 2010, titled “*Ovarian cancer development and metastasis*” *(* 8). This article mentioned that the biological characteristics of ovarian cancer are different from other hematogenous metastases, and peritoneal dissemination is the main mode of metastasis. Ovarian cancer cells can adhere to the peritoneum and settle down, eventually leading to peritoneal metastasis. At present, the bursts of seven studies are still not over in which two studies each reviewed the epidemiology, pathogenesis, and treatment advances of ovarian and peritoneal cancers. Besides, WonJae Lee found that neutrophils have a negative effect on the peritoneal environment, promoting ovarian cancer metastasis (9). Andrew Chow from Weill Cornell Medical College found that peritoneal macrophages had an effect on the adaptive immune function of CD8^+^ T cells (10). Benjamin Izar (11) utilized single-cell RNA sequencing to analyze cells from ascites specimens of patients with high-grade serous ovarian cancer, creating a detailed single-cell landscape. This work laid the foundation for immunotherapy treatments for ovarian cancer.

### Hotspots and frontiers

Hotspots and frontiers are determined by cluster analysis of common keywords and burst keywords. Base on the above, we summarized the frontiers and hotspots of TME research in PM:

#### Cytoreductive surgery

CRS was first proposed in the 1980s by Paul Sugarbaker’s team at the Cancer Institute in Washington. CRS involves removing all visible peritoneal tumors through peritoneectomy combined with multiorgan resection and remains the cornerstone of peritoneal cancer treatment. Meta-analyses have confirmed that a 10% increase in maximum cell reduction corresponds to a 5.5% increase in median survival. For gastric cancer with peritoneal metastasis, complete CRS implies long survival and potential cure (12). The GASTRIPEC-I study demonstrated that the progression-free survival (PFS) and distant metastasis-free survival were significantly better in the CRS plus hyperthermic intraperitoneal chemotherapy (HIPEC) group compared to CRS alone (13). Among patients with platinum-sensitive recurrent ovarian cancer, the median overall survival (OS) was 61.9 months for those receiving complete CRS before platinum-based chemotherapy, compared with 27.7 months for incomplete CRS and 46.0 months for those not receiving CRS, with significant differences among the three cohorts (14). Among colorectal cancer (CRC) patients, the median OS for those who received CRS exceeded 40 months, although about three-quarters also received perioperative systemic chemotherapy. This survival advantage remains notable compared with 16–24 months of OS observed with systemic chemotherapy alone (15, 16). Similar outcomes were also observed in pseudomyxoma peritonei(PMP) and diffuse malignant pleural mesotheliona (DMPM) patients. An international series showed that patients undergoing complete CRS(CC-0) had significantly better survival compared to those with incomplete CRS (CC-1) for DMPM (17). Likewise, among 738 PMP patients who underwent complete CRS (CCRS), the 10-year survival rate was 70.3%, compared to 8.1% among 242 patients receiving maximal tumor debulking (18). In addition, complete CRS improves survival even in patients with peritoneal metastases originating from rare primary sites, including pancreatic, biliary, breast and lung cancers, neuroendocrine tumors, or sarcomas (19). It is worth noting that most studies referenced above involve CRS combined with intraperitoneal chemotherapy rather than CRS alone. Currently, CRS combined with intraperitoneal chemotherapy is considered the preferred treatment option for PM.

At present, there is no standardized definition of PM resectability, and a comprehensive judgment is made based on patients and tumors characteristics. The peritoneal cancer index (PCI) serves as a useful tool for assessing the feasibility of CRS. Studies have shown that each point increase in PCI corresponds to a 5% decrease in 5-year survival (20). Imaging modalities such as magnetic resonance imaging (MRI), positron emission tomography-computed tomography (PET-CT) with the tracer 18F-fluorodeoxyglucose (18F-FDG), and other imaging methods have also been explored to evaluate the resectable peritoneal metastases preoperatively, with some progress. However, surgical exploration remains the gold standard for confirm the feasibility of complete CRS (21).

#### Intraperitoneal chemotherapy

Due to the presence of the plasma-peritoneal barrier, the clearance rate of chemotherapy drugs in the abdominal cavity is significantly slower than in systemic chemotherapy, resulting in a higher local drug concentrations in the peritoneum. HIPEC is the most widely used form of intraperitoneal therapy. Its efficacy is influenced by multiple factors, including chemotherapy drug dose, duration, intraperitoneal pressure level, carrier solution, leading to variations across institutions (22). A retrospective study demonstrated that the median survival of PMP patients treated with CRS combined with HIPEC was 56 months, compared to 23 months for those receiving CRS alone. CRS combined with HIPEC significantly improved the five-year OS in PMP patients (57.8% vs 46.2%) (23). The OVIHIPEC-1 study showed that CRS plus HIPEC prolonged OS by 12 months in advanced ovarian cancer (24). Similarly, CRS-HIPEC has been proven superior to CRS alone for gastric cancer peritoneal metastasis, with consensus among Chinese and Western scholars (25). However, as previously mentioned, HIPEC efficacy varies. The PRODIGE 7 trial results showed that CRS combined with high-dose oxaliplatin HIPEC did not benefit patients with colorectal cancer peritoneal metastasis, and the incidence of ≥3 postoperative complications was significantly increased (15).

Pressurized peritoneal aerosol chemotherapy (PIPAC) is a new intraperitoneal chemotherapy method, first introduced in 2011. Using a standard peritoneal pressure of 12mmHg, chemotherapy drugs are aerosolized and delivered via atomizing devices, typically during laparoscopic exploration. Compared to HIPEC, PIPAC offers clear advantages, such as less surgical trauma, higher local drug concentration, fewer systemic side effects, repeatability, and real-time monitoring of peritoneal metastases (26, 27). Several studies have demonstrated PIPAC’s efficacy in CRC and gastric cancer patients (25, 28). Common chemotherapy agents used include cisplatin, doxorubicin, and oxaliplatin (29, 30). New innovations have emerged, such as electrostatic precipitation pressurized intraperitoneal aerosol chemotherapy (ePIPAC) (31), which uses electrostatic forces to achieve more uniform drug distribution and greater tissue penetration, and hyperthermic PIPAC (hPIPAC), where cisplatin is delivered at 38.8–40.2°C (32). However, the effectiveness and safety of these new techniques require further validation. Preclinical studies suggest that mild hyperthermia can inhibit tumor growth, stimulate vascular perfusion, upregulate vascular adhesion molecules, and promote immune cell infiltration, potentially influencing the peritoneal immune environment (33).

#### Immunotherapy

The peritoneal cavity harbors various immune cells including mononuclear/macrophages (CD68^+^), T lymphocytes, natural killer (NK) cells, dendritic cells (DCs). However, PM is often associated with immune evasion, leading to poor prognosis (34). Although immunotherapy has traditionally been considered ineffective for PM, recent studies suggest that activating local immune cells may offer therapeutic benefits.

Yuko Kumagai et al. established a mouse model of gastric cancer peritoneal metastasis and found that anti-programmed cell death protein 1 (PD-1) antibodies reduced mesenteric metastases by 30-40% through both intravenous and intraperitoneal administration. Additionally, CD8+ T cell density increased, while myeloid-derived suppressor cells (MDSC) density decreased within peritoneal tumors (35). Yu Seong Lee et al. found that combining oncolytic vaccinia virus and immune checkpoint inhibitors (ICIs) synergistically inhibited colon cancer peritoneal metastasis and malignant ascites (36). Other studies shown that combining anti-PD-1 and glucocorticoid-induced tumor necrosis factor receptor family-related protein (GITR) monoclonal antibodies significantly inhibit peritoneal lesions in ovarian cancer, with one-fifth of mice not developing metastases, whereas monotherapy showed minimal effects (37). A multicenter clinical study involving 502 metastatic CRC patients found that dual ICI significantly prolonged survival. However, ICI monotherapy was less effective in patients with PM and ascites (38). This suggests that immune microenvironment changes may render ICI monotherapy ineffective, and combination therapies may be more beneficial. MOC31PE, targeting the epithelial cell adhesion molecule (EpCAM), can elevate interleukin-6 (IL-6), IL-1, and tumor necrosis factor (TNF) levels, inducing immunogenic cell death and potentially controlling peritoneal metastasis (39). Clinical studies have shown that catumaxomab, targeting EpCAM and CD3, effectively controlled malignant ascites in gastric and ovarian cancers (40). Cancer vaccines, including cellular, viral vector, and molecular types, have recently shown progress in managing peritoneal carcinomatosis (PC). Yue-Qin Ai et al. found that after DC vaccines combined with intrapitoneal injection of cytokine-induced killer (CIK) cells increased CIK cells and decreased CD4+CD25+ regulatory T (Treg) cells, achieving a clinical remission rate of 40.9% and a disease control rate of 77.3% (41). In addition, IL-12 combined with an oncolytic virus reduced colon cancer peritoneal metastases in mice by activating NK cells and enhancing DC recruitment through IFN secretion (42). Preclinical studies indicated that intraperitoneal administration of anti-carcinoembryonic antigen (CEA) chimeric antigen receptor (CAR)-T cells provided better efficacy against colon cancer peritoneal metastasis than systemic administration. Furthermore, combining anti-CEA CAR-T therapy with anti-programmed death-ligand 1 (PD-L1) or anti-granulocyte receptor 1 (GR1) antibodies further enhanced therapeutic effects, suggesting a promising role for CAR-T therapy in PM treatment (43).

Reviewing the keyword timeline, the importance of immunotherapy has continued to grow relative to intraperitoneal chemotherapy and other hotspots, coinciding with the rise in immunotherapy-related publications. A further search of ClinicalTrials.gov (https://clinicaltrials.gov/) was conducted to summarize clinical studies of immunotherapy for peritoneal cancer. Inclusion criteria were: condition or disease as peritoneal cancer, other terms as immunotherapy, study type as interventional study, status as completed, and availability of results. Five records were initially screened, and three studies were ultimately eligible after manual review. All three studies were conducted in the United States and involved diseases such as peritoneal mesothelioma, ovarian, fallopian tube, or primary peritoneal cancer. Interventions included PD-1 monoclonal antibodies (nivolumab and pembrolizumab), cytotoxic T-lymphocyte-associated protein 4 (CTLA-4) monoclonal antibody ibritumomab, and DC vaccines, all demonstrating good anti-tumor effects (**Table 7**).

**Table 7:** Clinical studies related to immunotherapy in PM.

Rank	NCT Number	Official Title	Conditions	Intervention	Phases	Date	Country
1	NCT05041062	A Phase II Prospective, Open-label Trial of Perioperative Combination Nivolumab and Ipilimumab in Patients With Resectable Malignant Peritoneal Mesothelioma	Mesotheliom/ Peritoneal Mesothelioma	Drug: Nivolumab/ Drug: Ipilimumab	Phase2	2021/12/1-2023/4/13	USA
2	NCT03029598	Anti-PD-1 Therapy in Combination With Platinum Chemotherapy for Platinum Resistant Ovarian, Fallopian Tube, and Primary Peritoneal Cancer	Recurrent Fallopian Tube Carcinoma/ Recurrent Ovarian Carcinoma/ Recurrent Primary Peritoneal Carcinoma	Drug: Carboplatin/ Other: Laboratory Biomarker Analysis/ Biological: Pembrolizumab	Phase1 Phase2	2017/3/14-2021/12/31	USA
3	NCT02151448	A Phase 1/2 Trial Evaluating αDC1 Vaccines Combined With Tumor-Selective Chemokine Mod ulation as Adjuvant Therapy After Surgical Resection of Peritoneal Surface Malignancies	Malignant Neoplasm of Pancreas Metastatic to Peritoneal Surface/ Peritoneal Carcinomatosis/ Malignant Peritoneal Mesothelioma	Biological: DC vaccine/ Drug: Celecoxib/ Drug: Interferon Alfa-2b/ Biological: rintatolimod	Phase1 Phase2	2014/7/1-2019/2/18	USA

#### TAMs

TAM are the most abundant immune cells in the TME. Among them, M2 macrophages can induce angiogenesis, secrete IL-10 and TNF-α, upregulate PD-L1 expression on cytotoxic T cells, and promote the infiltration of Treg cells, thus causing the pre-metastatic niche and immune escape (44). In PM with malignant ascites, increased secretion of IL-6 and IL-10 increases promotes the polarization of peritoneal macrophages to M2 macrophages (45). Peritoneal macrophages also exhibit oxidative phosphorylation, leading to reduced CD8+ T cell infiltration and suppression of adaptive immunity (46). Therefore, targeting TAMs represents a potential research hotspot in PM.

A. E. Ryan et al. found that targeting nuclear factor kappa B (NF-κB) in CT26 colon cancer cells induced macrophages polarization to the M1 type, thereby alleviating peritoneal metastasis (47). Gemcitabine (GEM) has demonstrated immunomodulatory effects in pancreatic cancer patients. Animal experiments confirmed that GEM induced M1-like polarization of peritoneal macrophages in a reactive oxygen species (ROS)-dependent manner, thereby inhibiting metastasis (48). Hydroxygenkwanin has also been shown to promote macrophage polarization towards M1 phenotype by activating p-NF-κB signaling, thus inhibiting peritoneal metastasis (49). Since immune escape in colorectal cancer is associated with macrophage-mediated clearance of apoptotic cells, researchers have proposed an RNA therapy approach to selectively inhibit macrophage-mediated endocytosis in the TME as a potential treatment strategy (50).

#### CAFs

Higher expression of CAFs in the TME can stimulate M2 macrophage migration and is associated with poor prognosis in gastric cancer and peritoneal metastasis (51). Compared to normal gastric fibroblasts (NGFs), CAFs increase IL-6 secretion and induce chemotherapy resistance. Studies have shown that peritoneal administration of paclitaxel combined with OBP-702 synergistically inhibits peritoneal metastasis and reduces the number of CAFs in peritoneal metastasis (52). CAFs are also closely linked to peritoneal metastasis of ovarian cancer and constitute key components of metastatic niche. MiR-29c-3p, present in exosomes from retinal CAFs, co-mediates peritoneal metastasis by acting on matrix metalloproteinase 2 (53). GLIS1 overexpression in CAFs has also been found to promote metastasis and may serve as a potential therapeutic target (54). Studies have indicated that key enzymes involved in fatty acid oxidation (FAO) are downregulated in colorectal cancer patients with PM. CAFs promotes the proliferation, migration and invasion of colon cancer cells by enhancing glycolysis (55). Clinical studies have identified CAFs co-expressed CD70 and periostin (POSTN) as associated with advanced pT stage in colon cancer or peritoneal metastasis, suggesting a promising therapeutic target (56).

#### Endothelial growth factor

Vascular endothelial growth factor (VEGF) has been closely associated with tumor progression (57). Gastric cancer cells are known to highly express EGF mRNA, and intraperitoneal injection of bevacizumab significantly inhibited peritoneal metastasis and reduced malignant ascites in tumor-bearing mice (58). Patients with gastric cancer and high ascites VEGF levels exhibited poor survival outcomes. A chemotherapy-resistant patient with malignant ascites experienced an eight-month survival benefit from bevacizumab (59). Zinc protoporphyrin IX, an antiangiogenic drug, has also been shown to inhibit gastric cancer peritoneal metastasis and prolong survival in mice (60). VEGF levels are significantly elevated in malignant ascites (MA) of gastric cancer, resulting in enhanced cell adhesion. Studies have confirmed that blocking VEGF eliminates the ability of MA to stimulate gastric cancer cells adhesion to human peritoneum and subsequent metastasis (61). In ductal pancreatic cancer, high VEGF expression was significantly associated with liver metastasis, but no clear association with peritoneal metastasis was observed (62). Further research is needed to confirm this relationship.

### Comparative perspectives across cancer types

In addition, the frontiers and hotspots of the TME have been explored in lung (63), colorectal (64), hepatocellular (65), breast (66, 67), pancreatic (68), hematological malignancies (69) and cervical cancer (70) through bibliometric studies. By keyword analysis, five studies identified immunotherapy as a future research direction, three studies highlighted the critical role of CAFs in regulating the TME, and one study mentioned macrophage polarization. These findings are consistent with the hotspots identified in our study. Other emerging directions, including new technologies, ferroptosis, biomarkers, and nanoparticles, are also worthy of exploration in peritoneal cancer. In terms of methodology, nearly all studies were restricted to English publications, with VOSviewer, CiteSpace, and the R package ‘bibliometrix’ being the most commonly used visualization tools. China and the USA were the countries with the highest number of publications. Related studies were more frequently published in Frontiers in Immunology, Frontiers in oncology, and Cancer. Thus, we conclude that the immune microenvironment is the most critical component of the TME, closely linked to the treatment of most malignancies, and will remain a research hotspot for the foreseeable future.

### Limitations

Although this study followed the principles of bibliometric analysis, several potential limitations exist. First, the analysis was restricted to English-language publications, which may have introduced publication bias. Nevertheless, given the relatively small proportion of non-English studies, the WoSCC still captures the majority of relevant research, ensuring the representativeness of our findings. Second, analyses were limited to articles and reviews published in WoSCC, and articles included in other databases (e.g., PubMed and Scopus) should be followed up jointly to avoid omissions. Third, the literature search concluded in August 2024, which may have resulted in missing newly published studies during the subsequent period. Additionally, some recent high-quality studies may have been overlooked due to their low citation counts. Finally, bibliometric analysis is limited to metadata and does not involve full-text examination, potentially missing significant insights such as authors’ perspectives and future research directions.

## Conclusion

Through bibliometric research, we found that research on the TME in PM has garnered increasing attention, with annual publications and citations rising steadily. Thus, the TME of PM will continue to be an active research field. Based on publication output, citation counts, and H-index, the United States, China, and Japan are the most influential countries. Strengthening international collaboration remains essential. Cancer and Oncogene are the most influential journals, and strong connections exist among different journals. Keyword cluster analysis suggests that the main research trend of TME in PM focuses on CRS combined with various forms of intraperitoneal chemotherapy, which has been validated by high-quality studies. Intraperitoneal chemotherapy technologies continue to advance, offering patients more treatment options. Notably, immunotherapy is poised to become the most significant and enduring area of research in the future. In addition, TAMs, CAFs, and epidermal growth factor in the TME have been intensively studied, presenting promising therapeutic targets and emerging research frontiers. This study summarizes the major hotspots in the TME of PM and may contribute meaningfully to the advancement of treatment strategies for PM.

## Data Availability

The original contributions presented in the study are included in the article/Supplementary Material. Further inquiries can be directed to the corresponding author.
